# Incidence of complications and urinary incontinence following endoscopic enucleation of the prostate in men with a prostate volume of 80 ml and above: results from a multicenter, real-world experience of 2512 patients

**DOI:** 10.1007/s00345-024-04886-6

**Published:** 2024-03-20

**Authors:** Vineet Gauhar, Daniele Castellani, Thomas R. W. Herrmann, Mehmet Ilker Gökce, Khi Yung Fong, Nariman Gadzhiev, Vigen Malkhasyan, Giacomo Maria Pirola, Angelo Naselli, Abhay Mahajan, Pankaj Nandkishore Maheshwari, Sarvajit Biligere, Azimdjon N. Tursunkulov, Furkat Nasirov, Vladislav Petov, Marco Dellabella, Ee Jean Lim, Moisés Rodríguez Socarrás, Marek Zawadzki, Luigi Cormio, Gian Maria Busetto, Jeremy Yuen-Chun Teoh, Bhaskar Kumar Somani, Dmitry Enikeev, Mario Sofer, Fernando Gómez Sancha

**Affiliations:** 1 Department of Urology, Ng Teng Fong General Hospital, Singapore, Singapore; 2 Urology Unit, IRCCS INRCA, Ancona, Italy; 3 Urology Unit, Azienda Ospedaliero-Universitaria delle Marche, Università Politecnica delle Marche, Via Conca 71, 60126 Ancona, Italy; 4 Department of Urology, Kantonspital Frauenfeld, Spital Thurgau AG, Frauenfeld, Switzerland; 5 Department of Urology, Ankara University School of Medicine, Ankara, Turkey; 6 Yong Loo Lin School of Medicine, National University of Singapore, Singapore, Singapore; 7 Department of Urology, Saint-Petersburg State University Hospital, Saint-Petersburg, Russian Federation; 8 Urology Unit, A.I. Evdokimov Moscow State University of Medicine and Dentistry, Moscow, Russian Federation; 9 Urology Unit, San Giuseppe Hospital, IRCCS Multimedica, Multimedica Group, Milan, Italy; 10 Sai Urology Hospital and Mahatma Gandhi Mission’s Medical College and Hospital, Aurangabad, India; 11 Urology Unit, Fortis Hospital Mulund, Mumbai, India; 12 Urology Division, AkfaMedline Hospital, Tashkent, Uzbekistan; 13 Institute for Urology and Reproductive Health, Sechenov University, Moscow, Russian Federation; 14 Department of Urology, Singapore General Hospital, Singapore, Singapore; 15 Department of Urology and Robotic Surgery, ICUA-Clínica CEMTRO, Madrid, Spain; 16 Urology Unit, St. Anna Hospital, Piaseczno, Poland; 17 Andrology and Urology Unit, Bonomo Teaching Hospital, Andria, Italy; 18 Department of Urology, Ospedali Riuniti di Foggia, University of Foggia, Foggia, Italy; 19 S.H. Ho Urology Centre, Department of Surgery, The Chinese University of Hong Kong, Hong Kong, China; 20 Department of Urology, University Hospitals Southampton, NHS Trust, Southampton, UK; 21 Department of Urology, Medical University of Vienna, Vienna, Austria; 22 Urology Department of Urology, Rabin Medical Center, Petah Tikva, Israel; 23 Department of Urology, Tel-Aviv Sourasky Medical Center, Sackler Faculty of Medicine, Tel-Aviv University, Tel-Aviv, Israel

**Keywords:** Prostatic hyperplasia, Endoscopic enucleation of the prostate, Laser therapy, Postoperative complications, Urinary incontinence

## Abstract

**Purpose:**

To evaluate complications and urinary incontinence (UI) after endoscopic enucleation of the prostate (EEP) stratified by prostate volume (PV).

**Methods:**

We retrospectively reviewed patients with benign prostatic hyperplasia who underwent EEP with different energy sources in 14 centers (January 2019–January 2023). Inclusion criteria: prostate volume ≥ 80 ml. Exclusion criteria: prostate cancer, previous prostate/urethral surgery, pelvic radiotherapy.

Primary outcome: complication rate. Secondary outcomes: incidence of and factors affecting postoperative UI. Patients were divided into 3 groups. Group 1: PV = 80–100 ml; Group 2 PV = 101–200 ml; Group 3 PV > 200 ml. Multivariable logistic regression analysis was performed to evaluate independent predictors of overall incontinence.

**Results:**

There were 486 patients in Group 1, 1830 in Group 2, and 196 in Group 3. The most commonly used energy was high-power Holmium laser followed by Thulium fiber laser in all groups. Enucleation, morcellation, and total surgical time were significantly longer in Group 2. There was no significant difference in overall 30-day complications and readmission rates. Incontinence incidence was similar (12.1% in Group 1 vs. 13.2% in Group 2 vs. 11.7% in Group 3, *p* = 0.72). The rate of stress and mixed incontinence was higher in Group 1. Multivariable regression analysis showed that age (OR 1.019 95% CI 1.003–1.035) was the only factor significantly associated with higher odds of incontinence.

**Conclusions:**

PV has no influence on complication and UI rates following EEP. Age is risk factor of postoperative UI.

**Supplementary Information:**

The online version contains supplementary material available at 10.1007/s00345-024-04886-6.

## Introduction

Following its inception in 1983, endoscopic enucleation of the prostate (EEP) has continuously advanced and has gained popularity and acceptance among urologists, primarily due to the use of bipolar energy and lasers and the introduction of morcellators [1]. EEP, particularly holmium laser enucleation of the prostate (HoLEP), is touted as a size-independent procedure with a low morbidity rate [2]. In large (80–150 ml) [3] and very large prostates (> 150/200 ml) [4, 5], HoLEP showed an acceptable rate of complications with excellent functional outcomes. Nevertheless, postoperative urinary incontinence is one of the most concerning complications that affect the quality of life for patients undergoing surgery for benign prostatic hyperplasia [6]. Currently, data comparing complications and incontinence rates following EEP with different laser energies in men with a prostate volume larger than 80 ml and stratified by prostate volume are lacking.

The primary aim of this study is to investigate the complication rates after EEP from a multicenter, real-world experience when different laser energies are used for EEP comparing patients with different prostate volumes. The secondary outcomes are to assess the incidence of and factors affecting postoperative urinary incontinence.

## Materials and methods

We performed a retrospective analysis of BPH patients who underwent EEP in 14 centers between January 2019 and January 2023. Inclusion criteria were prostate volume equal to or above 80 ml, lower urinary tract symptoms not responding to or worsening despite medical therapy acute urinary retention, recurrent urinary tract infections or hematuria due to BPH, and bilateral hydronephrosis with renal impairment. Exclusion criteria were prostate cancer, previous prostate/urethral surgery, and pelvic radiotherapy. Concomitant bladder lithotripsy was allowed. Prostate cancer was ruled out before EEP in patients with elevated PSA or when clinically suspected by performing a prostate biopsy. At baseline, the following data were gathered: age, American Society of Anesthesiologists (ASA) score, presence of a preoperative indwelling catheter, International prostate symptom score (IPSS) with quality of life (QL) item, PSA, post-void residual urine (PVR), and maximum flow rate (Qmax) at uroflowmetry. Thirteen surgeons with previous experience of at least 200 laser EEP were involved in all procedures. Prostate volume was measured by transrectal ultrasonography. Patients taking oral anticoagulants at baseline were switched to low-weight molecular heparin in preparation for surgery and resumed as per each center's discretion, whilst single antiplatelet agents were maintained. All patients received antibiotic prophylaxis following local protocols.

Energy choice and EEP technique were at the surgeon’s discretion based on their experience and available resources. Morcellation was performed in all cases after enucleation. A catheter sized 20 Ch or 22 Ch was placed in the bladder after the procedure's completion, and continuous irrigation was maintained until the urine cleared. Enucleation time was considered as the time from the beginning of the enucleation until the start of morcellation. Surgical time encompassed the period from cystoscopy to catheter placement.

Patients were divided into three groups based on the prostate volume. Group 1 consisted of patients who had a prostate volume between 80 and 100 ml; Group 2 included patients who had a prostate volume between 101 and 200 ml; Group 3 included patients with a prostate volume above 200 ml. Complications were divided into early (within 30 days of surgery) and late. Early complications were graded according to the modified Clavien–Dindo classification. Urinary incontinence was defined as any complaint of urine leakage according to patient reports and classified into three types: (i) urge incontinence: involuntary loss of urine associated with urgency; (ii) stress incontinence: involuntary loss of urine on effort or physical exertion, or on sneezing or coughing; and (iii) mixed incontinence: both stress and urgency urinary incontinence [7]. To evaluate the duration of incontinence, we categorized it into three groups depending on the time between catheter removal and when patients reported that their incontinence had stopped: less than 1 month, 1–3 months, and more than 3 months. The maximum follow-up was 1 year.

Institutional board review approval was obtained by the leading center (Asian Institute of Nephrology and Urology, AINU #11/2022), and the remaining centers had approvals from their Institutional boards. All patients signed an informed consent to collect their de-identified data.

### Statistical analysis

Continuous variables were assessed for their normal distribution with the Kolmogorov–Smirnov test and are reported as median and interquartile range. Categorical variables are reported as absolute frequency and percentage. For between-group comparisons, the *χ*^2^ test for categorical parameters and the Kruskal–Wallis test for continuous variables were utilized. A multivariable logistic regression analysis was performed to evaluate factors associated with overall postoperative incontinence. Variables available for all patients were entered into the multivariable model to assess their significance as independent predictors. Predictors were described using odds ratios (OR), 95% confidence intervals (CI), and *p* values. A two-tailed *p* value < 0.05 was considered significant. All statistical tests were performed using R Statistical language, version 4.3.0 (R Foundation for Statistical Computing, Vienna, Austria).

## Results

During the study period, 2512 patients met the inclusion criteria and were included in the analysis. Supplementary Table S1 shows participating centers with their number of included patients. Among them, there were 486 patients in Group 1, 1830 in Group 2, and 196 in Group 3. Patient baseline characteristics are presented in Table 1. Patients in Group 1 were significantly older [71.0 (66.0–76.0) vs. 69.0 (63.0–74.0) vs. 68.0 (61.0–73.0), *p* < 0.001]. There was a significantly higher proportion of patients with a preoperative indwelling catheter in Group 1 (28.2% vs. 18.7% vs. 9.7%). There was no difference in preoperative Qmax, whilst IPSS was significantly higher in Group 3 (26 vs. 25 in Group 2 vs. 23 in Group 1, *p* < 0.001). PSA was significantly higher in Group 3 [5.5 (3.80–7.90) ng/ml vs. 4.47 (2.90–6.70) ng/ml in Group 2 vs. 4.30 (2.60–7.25) ng/ml in Group 1].

**Table 1:** Baseline characteristics of all patients and according to prostate volume

	*n* with data available	All patients (*n* = 2512)	Group 1	Group 2	Group 3	*p*
			80–100 ml (*n* = 486)	101–200 ml (*n* = 1830)	> 200 ml (*n* = 196)	
Age, years, median (IQR)	2512	69.00 (64.00, 74.55)	71.00 (66.00, 76.00)	69.00 (63.00, 74.00)	68.00 (61.00, 73.00)	** < 0.001**
ASA score, *n* (%)	1997					** < 0.001**
1		483 (24.2)	61 (14.1)	401 (28.1)	21 (15.1)	
2		989 (49.5)	223 (51.5)	680 (47.7)	86 (61.9)	
3		503 (25.2)	143 (33.0)	332 (23.3)	28 (20.1)	
4		22 (1.1)	6 (1.4)	12 (0.8)	4 (2.9)	
Preoperative indwelling catheter for acute urinary retention, *n* (%)	2512	498 (19.8)	137 (28.2)	342 (18.7)	19 (9.7)	** < 0.001**
Preoperative IPSS, median (IQR)	1834	24.00 (22.00, 28.00)	23.00 (20.00, 26.00)	25.00 (22.00, 28.00)	26.00 (23.00, 29.00)	** < 0.001**
Preoperative QOL, median (IQR)	1827	5.00 (4.00, 5.00)	5.00 (4.00, 5.00)	5.00 (4.00, 5.00)	5.00 (4.00, 5.00)	**0.046**
Preoperative Qmax, ml/sec, median (IQR)	1616	8.00 (6.50, 10.00)	8.00 (6.55, 11.00)	8.00 (6.50, 10.00)	8.00 (7.00, 10.00)	0.724
Preoperative PVRU, median (IQR)	1554	70.00 (50.00, 98.00)	80.00 (52.50, 112.50)	70.00 (50.00, 100.00)	66.00 (57.50, 75.00)	**0.008**
Preoperative PSA, ng/ml, median (IQR)	1865	4.50 (3.00, 6.90)	4.30 (2.60, 7.25)	4.47 (2.90, 6.70)	5.50 (3.80, 7.90)	**0.001**

Bold value stands for significant *p* value

IQR, interquartile range; Qmax, maximum flow rate; QL, quality of life; IPSS, International Prostate Symptoms Score; PSA, prostate specific antigen; PVRU, post-voiding residual of urine; ASA, American Society of Anesthesiologists

Supplementary Table S2 shows intraoperative characteristics. The most commonly used energy for enucleation was high-power Holmium laser followed by Thulium fiber laser in all groups. Almost all procedures in each group were performed using a scope sized 26 or 27 Fr. There was a significantly higher usage of en-bloc enucleation in Group 1 (75.1% vs. 57.5% in Group 2 vs. 52.6% in Group 3, *p* < 0.001). The early apical release technique was employed in almost two-thirds of patients in each group but more frequently in Group 1 (79.6% vs. 71.9% vs. 73%, *p* = 0.003). Piranha morcellator (Richard Wolf, Knittlingen, Germany) was most frequently used in Group 1 (83.3%) and 2 (67.1%), whilst MultiCut Solo (Jena Surgical, Asclepion Laser Technologies, Jena, Germany) in Group 3 (50%). There was a significantly higher rate of bladder lithotripsy in Group 1 (9.7%) than in Group 2 (6.1%) and 3 (6.7%, *p* = 0.021). Enucleation, morcellation, and total surgical time were significantly longer in Group 2.

Table 2 shows postoperative outcomes, early and late complications, and incontinence rates. Median postoperative catheter time was 2 (1.0–2.0) days in the whole series with no difference among the groups. There was no significant difference in overall early postoperative complications and 30-day readmission rates among the groups. Overall, 86.1% of patients had no complications with a similar rate among the groups. No patients in Group 1 required either blood transfusion or surgical hemostasis of delayed secondary bleeding. Two patients in Group 2 died of complications from cardiovascular events. The late postoperative complication rate was also similar among the groups, with urethral stricture requiring dilation only the most common one (overall rate 0.8%).

**Table 2:** Postoperative outcomes and complications, and urinary incontinence

	*n* with data available	All patients (*n* = 2512)	Group 1	Group 2	Group 3	*p*
			80–100 ml (*n* = 486)	101–200 ml (*n* = 1830)	> 200 ml (*n* = 196)	
Early (30 days) complications, *n* (%)	2512					0.075
None		2162 (86.1)	437 (89.9)	1552 (84.8)	173 (88.3)	
Acute urinary retention (Clavien 2)		84 (3.3)	22 (4.5)	59 (3.2)	3 (1.5)	
Fluid extravasation (Clavien 2)		3 (0.1)	0 (0.0)	3 (0.2)	0 (0.0)	
Morcellation minor bladder injury (Clavien 1)		6 (0.2)	4 (0.8)	6 (0.3)	0 (0.0)	
Redo surgery within 30 days (Clavien 3)		1 (0.03)	0 (0.0)	1 (0.1)	0 (0.0)	
Prolonged irrigation for haematuria (Clavien 2)		79 (3.1)	0 (0.0)	70 (3.8)	5 (2.6)	
Blood transfusion (Clavien 2)		10 (0.4)	0 (0.0)	9 (0.6)	1 (0.5)	
Postoperative bleeding requiring surgical haemostasis (Clavien 3)		22 (0.9)	0 (0.0)	21 (1.4)	1 (0.5)	
Urinary tract infections (Clavien 2)		97 (3.9)	12 (2.5)	75 (4.1)	10 (5.1)	
Sepsis (Clavien 4)		6 (0.2)	2 (0.4)	4 (0.2)	0 (0.0)	
Secondary morcellation (Clavien 3)		18 (0.7)	3 (0.6)	12 (0.7)	3 (1.5)	
Ureteral orifice injury requiring stenting (Clavien 3)		4 (0.2)	0 (0.0)	4 (0.2)	0 (0.0)	
Cardiovascular complications (Clavien 4)		8 (0.3)	4 (0.8)	4 (0.2)	0 (0.0)	
Bulbar urethral stricture requiring urethrotomy (Clavien 3)		1 (0.03)	1 (0.2)	0 (0.0)	0 (0.0)	
Death for cardiovascular events (Clavien 5)		2 (0.08)	0 (0.0)	2 (0.11)	0 (0.0)	
30-day readmission, *n* (%)	2055	53 (2.6)	6 (3.6)	46 (2.7)	1 (0.5)	0.150
Postoperative catheter time (days), median (IQR)	2040	2 (1.00, 2.00)	2.00 (1.00, 2.00)	2.00 (1.00, 2.00)	2.00 (1.06, 2.00)	0.076
Postoperative incontinence, *n* (%)	2512	324 (12.9)	59 (12.1)	242 (13.2)	23 (11.7)	0.720
Urge		54 (17.5)	2 (3.6)	50 (21.5)	2 (9.5)	
Stress		192 (62.1)	48 (87.3)	131 (56.2)	13 (61.9)	
Mixed		63 (20.4)	5 (9.1)	52 (22.3)	6 (28.6)	
Duration of incontinence for those affected, *n* (%)	306					0.651
< 1 month		160 (52.3)	24 (44.4)	123 (53.5)	13 (59.1)	
1–3 months		97 (31.7)	19 (35.2)	71 (30.9)	7 (31.8)	
> 3 months		49 (16.0)	11 (20.4)	36 (15.7)	2 (9.1)	
Histology, *n* (%)	2109					** < 0.001**
Benign Prostatic Hyperplasia		1977 (93.7)	401 (90.7)	1432 (94.3)	144 (96.6)	
Incidental prostate cancer		117 (5.5)	41 (9.3)	71 (4.7)	5 (3.4)	
Prostatic intraepithelial neoplasia		15 (0.7)	0 (0.0)	15 (1.0)	0 (0.0)	
Delayed (> 30 days) complications, *n* (%)	2055					0.606
None		2012 (97.9)	165 (97.6)	1664 (97.8)	183 (98.9)	
Urethral stricture requiring dilation only		17 (0.8)	3 (1.8)	14 (0.8)	0 (0.0)	
Urethral stricture requiring urethrotomy		14 (0.7)	1 (0.6)	11 (0.6)	2 (1.1)	
Bladder neck stenosis requiring incision		10 (0.5)	0 (0.0)	10 (0.6)	0 (0.0)	
Stress incontinence requiring sling		2 (0.1)	0 (0.0)	2 (0.1)	0 (0.0)	

Bold value stands for significant *p* value

Regarding overall postoperative incontinence, there was no significant difference among the groups regarding incidence (12.1% in Group 1 vs. 13.2% in Group 2 vs. 11.7% in Group 3, *p* = 0.72). Interestingly, the rate of stress and mixed incontinence was higher in Group 1 than in other ones. Yet, there was no significant difference in the duration of incontinence even if the rate of incontinence after 3 months was higher in Group 1 (20.4% vs. 15.7% vs. 9.1%).

At multivariable analysis, age (OR 1.019 95% CI 1.003–1.035) was the only factor significantly associated with higher odds of incontinence, whilst high-power Holmium laser (OR0.468 95% CI 0.257–0.889) and Virtual basket (OR 0.123 95% CI 0.027–0.398) were factors significantly associated with lower odds of being incontinent (Supplementary Table S3).

## Discussion

EAU, NICE, and AUA guidelines recommend laser-based EEP for prostates larger than 80 ml a reference standard being currently held for large prostates [8]. Recently, laparoscopic or robotic simple prostatectomy has been advocated for prostates > 80 cc with pros and cons for each [9] but even for large prostates HoLEP is as safe and equally effective and offers shorter hospitalizations, lower transfusion rates, shorter catheterization time, lower costs, and even feasible for same-day discharge [10]. However, most of the published studies included single-center series with the majority of the large prostate volumes ranging between 80 and 100 ml. Even fewer studies report outcomes for prostate volumes of more than 150–200 ml [5, 11, 12]. Glybochko et al. also classified 458 patients into 3 categories akin to ours but had only 12 cases with a prostate volume > 200 ml and 169 in the 100–200 ml group [12]. In our series for the first time we report that irrespective of size, any laser and even electrical energy can be used for EEP making it a size and energy-independent procedure. However, monopolar enucleation was not much favored as it was only reported in 10 patients from one center and restricted to patients with prostates < 200 ml. Indeed, a recent analysis of 4512 patients from REAP registry comparing high-power HoLEP with ThuFLEP for median prostate volume of 80 ml, shows that early and delayed outcomes of enucleation with ThuFLEP are comparable to those with HoLEP, with similar improvements in micturition parameters and IPSS [13].

In our global multicenter study, perhaps for the first time, we also can show that when EEP is performed by experienced surgeons complication and incontinence rates are equivalent irrespective of using an en-bloc or non-en-bloc technique with the former being the more preferred approach for all prostate sizes. This may be in part related to the fact that in large prostates, en-bloc EEP is a safe technique that allows for easier recognition of the surgical plane, a key factor that determines the progress of the surgery [14]. Limited data is available for ThuFLEP for large prostates but Enikeev et al. have shown that compared to open prostatectomy, ThuFLEP is a minimally invasive modality associated with shorter hospital stay, a significantly greater return to normal activities, and a considerable reduction in rehabilitation time [15]. In our series as well after HoLEP, ThuFLEP was the next most preferred choice in 18.7% of cases. Our series, being more recent, concretely demonstrates the evidence of various EEP techniques, approaches, safety, and utility of enucleation even in large and very large prostates. This is another proof of concept that the statement EEP is a truly size-independent procedure once the learning curve is mastered [16], even if complications are a part of every surgery and EEP is no exception [17].

It has been reported that when tackling > 100 ml prostates surgery may be laborious, and time-consuming, extraction can be associated with marked hemorrhage often needing electrocautery, and often significant intravesical protrusion can lead to accidental ureteric orifice injury and sometimes prostates larger than 200 ml needed adenoma extraction by cystotomy [12]. Thus, a surgeon needs at least 20 procedures on prostates of 100 ml to attain a learning curve threshold to tackle the larger glands [12]. This is reflected in the enucleation and morcellation times and efficiency as well. Zell et al. in their series of 88 patients stratified the prostates into 200–299 ml (76 cases) and ≥ 300 ml (12 cases) and reported that mean operative and enucleation time were not different between the two groups but enucleation efficiency was significantly greater for glands ≥ 300 ml (2.6 cc/min vs. 2.0 cc/min, *p* = 0.04) [5]. Yet, morcellation time was longer in the ≥ 300 ml group (74.5 min vs. 46.8 min, *p* = 0.021). In our series, 86.1% of patients had no complications with a similar rate among the groups. However, post-operatively need for prolonged bladder irrigation for hematuria, (3.85%), bleeding needing surgical hemostasis (1.4%), and need for blood transfusion (0.6%) albeit low was all seen in Group 2 and was perhaps multifactorial attributed to shape and anatomical factors influencing dissection of the prostate, the efficacy of the laser energy devices used, technique, surgeon skill and patient factors [5, 11, 12, 17]. This is why the enucleation, morcellation, and total surgical time were significantly longer in Group 2. Moreover, this could reflect the fact that not all surgeons involved in our series are confident with very large prostates and probably surgeons performing EEP in prostate > 200 ml have better mastered the technique. Even ureteral orifice injury needing a double J stent was reported only in group 2. This complication typically happens in large prostates where the median lobe extends well beyond the trigone [12, 17]. As is seen in our series, patients with very large prostates must be counseled that at times the entire lobe cannot be extracted by sheer size or even inadvertently missed tissue may need secondary morcellation or even cystostomy and extraction [5, 17, 18]. Despite these reported complications, the size, laser type, and technique did not affect postoperative catheter time, overall early and late postoperative complications, and 30-day readmission rates among the groups in our series. Our findings may differ from other studies perhaps because this data is reflective of the surgeon's experience leading to a better understanding of technique and a careful pre and intraoperative approach in large and very large prostates. Also contributory is the availability of better surgical equipment as we can see many different energy sources, resectoscope sizes, and morcellators used in our study. Moreover, results might have also been influenced by the number of patients each center contributed reflecting surgeon’s expertise.

Concerning delayed complications, rates of urethral (1.5%) and bladder neck stenosis (0.5%) were within the literature incidence (1.7% and 0.66%, respectively) [19, 20].

Even in these large and very large prostates, incidental prostate cancer in our series (5.5%) was in line with the pooled incidence of 8% as reported in the literature [21].

Interestingly, incontinence rate, type, and duration did not differ between groups and prostate volume was not found to be associated with higher odds of incontinence but we found that aging was the only factor significantly associated with higher odds of incontinence. Therefore, elderly patients should be counseled preoperatively that they might experience postoperatively incontinence. We also found that the use of high power holmium laser and pulse modulation (i.e. virtual basket) were factors associated with lower odds of incontinence. This could be partially explained with faster enucleation, better identification of planes or achieving hemostasis allowed by high power holmium lasers [22]. Yet, minimizing the need for physical traction to identify planes or achieve hemostasis by virtue of pulse modulation could have further contributed in reducing urinary incontinence [23].

This study has some limitations. We do acknowledge that this is a retrospective study and there may be underreporting of complications. Yet, another limitation is the lack of data on preoperative incontinence. Furthermore, we did not collect data on pad test to evaluate the degree of incontinence and this is a further limitation. Finally, we are not able to provide more insight into the recovery of continence among incontinent patients because we did not gather data on incontinence treatments.

## Conclusion

Our real-world study reiterates that EEP is a safe, efficacious, and size-independent procedure. From a clinical perspective, laser EEP is preferred and all lasers can be used. Monopolar enucleation has probably phased out. Whilst en-bloc enucleation is probably best suited for prostates with more than 200 ml, any technique is feasible as per the surgeon's experience. Older men should be counseled regarding an increased possibility of higher odds of postoperative incontinence. Pulse modulated lasers could have the potential for minimizing urinary incontinence but this needs more evaluation.

## Supplementary Information

Below is the link to the electronic supplementary material.

Supplementary file 1 (DOCX 22 kb)

Supplementary file 2 (DOCX 22 kb)

Supplementary file 3 (DOCX 18 kb)

## Data Availability

Data are available on request from the authors.
