# Supplementary material for: Incidence of complications and urinary incontinence following endoscopic enucleation of the prostate in men with a prostate volume of 80 ml and above: results from a multicenter, real-world experience of 2512 patients
Source: World J Urol. 2024 Mar 20;42(1):180. doi: 10.1007/s00345-024-04886-6 (PMC10954849; doi:10.1007/s00345-024-04886-6)
Supplement: Supplementary file 1 — (DOCX 22 kb) [file 345_2024_4886_MOESM1_ESM.docx]

**Supplementary Table 1.** Centers where surgery was performed with included number of patients for each center.

| **Participant center** | **Number of included patients** |
| --- | --- |
| ICUA-Clínica CEMTRO, Madrid, Spain | 231 |
| St. Anna Hospital, Piaseczno, Poland | 93 |
| Saint-Petersburg State University Hospital, Saint-Petersburg, Russian Federation | 149 |
| A.I. Evdokimov Moscow State University of Medicine and Dentistry, Moscow, Russian Federation | 174 |
| Ankara University School of Medicine, Ankara, Turkey | 421 |
| Tel-Aviv Sourasky Medical Center, Sackler Faculty of Medicine, Tel-Aviv University, Tel-Aviv, Israel | 457 |
| IRCCS INRCA, Ancona, Italy | 73 |
| Sai Urology Hospital and Mahatma Gandhi Mission’s Medical College and Hospital, Aurangabad, India | 72 |
| AkfaMedline Hospital, Tashkent, Uzbekistan | 198 |
| Institute for Urology and Reproductive Health, Sechenov University, Moscow, Russian Federation | 391 |
| San Giuseppe Hospital, IRCCS Multimedica, Multimedica Group, Milan, Italy | 106 |
| Ospedali Riuniti di Foggia, University of Foggia, Foggia, Italy | 81 |
| Fortis Hospital Mulund, Mumbai, India | 30 |
| Ng Teng Fong General Hospital, Singapore, Singapore | 35 |
| **Total** | 2512 |
