# Supplementary material for: Incidence of complications and urinary incontinence following endoscopic enucleation of the prostate in men with a prostate volume of 80 ml and above: results from a multicenter, real-world experience of 2512 patients
Source: World J Urol. 2024 Mar 20;42(1):180. doi: 10.1007/s00345-024-04886-6 (PMC10954849; doi:10.1007/s00345-024-04886-6)
Supplement: Supplementary file 2 — (DOCX 22 kb) [file 345_2024_4886_MOESM2_ESM.docx]

**Supplementary Table 2.** Operative characteristics of all patients and according to prostate volume.

|  | N with data available | **All patients** (N=2512) | **Group 1**  80-100ml (N=486) | **Group 2**  101-200ml (N=1830) | **Group 3**  >200ml (N=196) | p |
| --- | --- | --- | --- | --- | --- | --- |
| Device energy, n (%)  Low-power Holmium laser  High-power Holmium laser*  Holmium laser with MOSES  Thulium fiber  Thulium-YAG  Bipolar enucleation  Monopolar enucleation  Virtual basket | 2512 | 80 (3.2)  1227 (48.8)  115 (4.6)  470 (18.7)  73 (2.9)  420 (16.7)  10 (0.4)  117 (4.7) | 4 (0.8)  351 (72.2)  28 (5.8)  41 (8.4)  23 (4.7)  30 (6.2)  0 (0.0)  9 (1.9) | 75 (4.1)  757 (41.4)  83 (4.5)  377 (20.6)  50 (2.7)  379 (20.7)  10 (0.5)  99 (5.4) | 1 (0.5)  119 (60.7)  4 (2.0)  52 (26.5)  0 (0.0)  11 (5.6)  0 (0.0)  9 (4.6) | **<0.001** |
| Scope size (Fr), n (%)  22  24  26  27 | 2512 | 53 (2.1)  142 (5.7)  2272 (90.4)  45 (1.8) | 31 (6.4)  41 (8.4)  405 (83.3)  9 (1.9) | 22 (1.2)  99 (5.4)  1673 (91.4)  36 (2.0) | 0 (0.0)  2 (1.0)  194 (99.0)  0 (0.0) | **<0.001** |
| Enucleation type, n (%)  3-lobe  2-lobe  En-bloc | 2512 | 304 (12.1)  687 (27.3)  1521 (60.5) | 27 (5.6)  94 (19.3)  365 (75.1) | 252 (13.8)  525 (28.7)  1053 (57.5) | 25 (12.8)  68 (34.7)  103 (52.6) | **<0.001** |
| Early apical release, n (%) | 2512 | 1845 (73.4) | 387 (79.6) | 1315 (71.9) | 143 (73.0) | **0.003** |
| Concomitant surgery for bladder stones, n (%) | 2512 | 174 (6.9) | 47 (9.7) | 112 (6.1) | 15 (7.7) | **0.021** |
| Total operation time, median [IQR]) | 2467 | 82.00 [57.00, 113.00] | 80.00 [60.00, 90.00] | 88.00 [55.00, 117.00] | 66.00 [52.29, 120.00] | **0.005** |
| Enucleation time, median [IQR]) | 1438 | 62.00 [33.00, 82.00] | 65.00 [40.00, 80.00] | 65.00 [32.50, 84.00] | 40.00 [32.75, 56.25] | **0.001** |
| Morcellation time, median [IQR]) | 1423 | 18.00 [12.00, 27.00] | 15.00 [10.00, 20.00] | 19.00 [12.00, 27.00] | 18.20 [16.00, 20.55] | **0.001** |
| Morcellator, n (%)  Cyber Blade  Hawk  MultiCut Solo  VersaCut  Piranha  Drillcut-X | 2460 | 70 (2.8)  1 (0.0)  423 (17.2)  48 (2.0)  1673 (68.0)  245 (10.0) | 5 (1.1)  1 (0.2)  0 (0.0)  3 (0.7)  383 (83.3)  68 (14.8) | 60 (3.3)  0 (0.0)  325 (18.0)  45 (2.5)  1210 (67.1)  164 (9.1) | 5 (2.6)  0 (0.0)  98 (50.0)  0 (0.0)  80 (40.8)  13 (6.6) | **<0.001** |
| Spinal anesthesia, n (%) |  | 1012 (40.3) | 117 (24.1) | 838 (45.8) | 57 (29.1) | **<0.001** |

**IQR:** interquartile range. Bold value stands for significant p value. *power >30 Watt
