# Supplementary material for: Incidence of complications and urinary incontinence following endoscopic enucleation of the prostate in men with a prostate volume of 80 ml and above: results from a multicenter, real-world experience of 2512 patients
Source: World J Urol. 2024 Mar 20;42(1):180. doi: 10.1007/s00345-024-04886-6 (PMC10954849; doi:10.1007/s00345-024-04886-6)
Supplement: Supplementary file 3 — (DOCX 18 kb) [file 345_2024_4886_MOESM3_ESM.docx]

**Supplementary Table 3.** Multivariable analysis of factors associated with overall urinary incontinence.

|  | **Multivariable analysis** | | |
| --- | --- | --- | --- |
|  | *OR 95% CI p* | | |
| Age | 1.019 | 1.003 – 1.035 | **0.021** |
| Prostate volume (vs 80-100ml) |  |  |  |
| 101-200 ml | 0.978 | 0.712 – 1.359 | 0.893 |
| >200 ml | 0.992 | 0.574 – 1.667 | 0.978 |
| Device energy (vs LP HL) |  |  |  |
| High-power Holmium laser | 0.468 | 0.257 – 0.889 | **0.016** |
| Holmium laser with MOSES | 1.062 | 0.521 – 2.200 | 0.870 |
| Thulium fiber laser | 0.602 | 0.295 – 1.258 | 0.169 |
| Thulium-YAG | 0.802 | 0.313 – 2.031 | 0.643 |
| Bipolar enucleation | 0.901 | 0.487 – 1.732 | 0.746 |
| Monopolar enucleation | 0.377 | 0.019 – 2.395 | 0.383 |
| Virtual basket | 0.123 | 0.027 – 0.398 | **0.002** |
| Enucleation type (vs 3-lobe) |  |  |  |
| 2-lobe | 1.063 | 0.684 – 1.664 | 0.789 |
| En-bloc | 0.711 | 0.483 – 1.062 | 0.089 |
| Early apical release | 1.105 | 0.711 – 1.723 | 0.659 |

Bold value stands for significant p value. **CI:** confidence interval. Bold value stands for significant p value. **LP HL**: Low-Power Holmium Laser. **YAG**: Yttrium-Aluminum-Garnet.
